# A 22-Year Study to Assess Disparities in Place of Death Among Patients With Diabetes

**DOI:** 10.7759/cureus.49929

**Published:** 2023-12-04

**Authors:** Zuhair S Siddiqui, Yingxia Xiao, Prince O Ansong, Suriya Senthilkumar Muthu, Angelina Sony, Shan Doghouz, Arjun Godavarthi

**Affiliations:** 1 Internal Medicine, Medical School, Lake Erie College of Osteopathic Medicine, Erie, USA; 2 Medicine, King Edward Medical University, Lahore, Lahore, PAK; 3 Internal Medicine, School of Medicine, University of Cape Coast, Cape Coast, GHA; 4 Internal Medicine, Saveetha Medical College and Hospital, Chennai, IND; 5 Family Medicine, Christian Medical College, Vellore, Vellore, IND; 6 Internal Medicine, K. S. Hegde Medical Academy, Mangalore, IND; 7 Internal Medicine, First Faculty of Medicine, Charles University, Prague, CZE; 8 Internal Medicine, Shadow Creek High School, Houston, USA

**Keywords:** home and hospice care, mortality trends, factors, predictors, diabetes mellitus

## Abstract

Background

This study examines disparities in the place of death in patients in the United States with diabetes mellitus (DM) using data from the CDC WONDER (Centers for Disease Control and Prevention’s Wide-Ranging Online Data for Epidemiologic Research) database covering a 22-year period (1999-2020). Looking at age, gender, ethnicity, and census location, among other variables, the study aims to understand trends and determinants of mortality at home or hospice care compared to mortality at a medical or nursing facilities.

Materials and methods

An online freely accessible mortality database, CDC WONDER database, was used to collect information regarding DM-related mortality, using the International Classification of Diseases, 11th Revision (ICD-11) code range E10-E14. To investigate patterns in location of death, the research population was split by census regions, racial categories, age groups, and gender. Statistical techniques such as univariate logistic regression and graphical representations were employed.

Results

Based on a study of 1,674,724 DM-related deaths, medical or nursing facilities recorded higher deaths (1,041,602) compared to home or hospice deaths (572,567). The highest number of deaths in home or hospice setting was recorded for the age group of 75-84 years (146,820), male gender (324,325), Census Region 3 (South) (225,636), and white race (458,690). Among the patients with death at home or a hospice center; the odds were highest for the age group of 55-64 years, male gender, Census Region 4 (West), and American Indian or Alaska Native race.

Discussion

The results showed a general upward trend in DM patients' deaths at home and in hospice care in the United States. Males, white people, and those in the age group of 75-84 years notably had the highest death rates. Regional differences also came into play, with the South showing the biggest trend in mortality. To better understand the underlying causes of these changes and to increase at-risk groups' access to healthcare facilities, more research is required.

Conclusion

There is an overall rising trend in home and hospice deaths in the United States for patients with DM, but with a steady dip between the years 2005 and 2010. Patient deaths from DM were categorized by age groups, gender, race, and census regions. The highest mortality trends are exhibited in whites, males, and those aged 75-84 years. Out of the census regions, the South has the highest mortality trend. Further studies could be carried out to determine the reasons for the rising trends in home or hospice deaths in the aforementioned groups and how to provide these groups with better access to healthcare facilities.

## Introduction

Death is a natural end to all life and we as humans are no exception. There has long been a stigma of fear attached to this process; however, efforts over the last few decades have been geared toward ensuring a seamless transition and alleviation of such fears. These efforts, within the United States, have included the establishment of hospice and palliative care as an institution backed by law and made accessible through Medicare as authorized by the U.S. Congress in 1982 [1]. Palliative care is defined as comfort care given with or without an intent of cure. It incorporates any treatment or intervention intended to alleviate pain or other debilitating symptoms. Hospice care, however, focuses solely on quality of life with no intent of cure. It is often provided in cases of terminal illness with a life expectancy of less than six months [2,3]. These modes of care can either be rendered in the comfort of people's homes or within an authorized institution. Diabetes mellitus (DM) is the most common endocrine disease in the United States. It is a chronic condition that affects the uptake of glucose in the body due to either the absence or insensitivity to insulin. DM affects more than 37 million adults in the USA and is the eighth leading cause of death [4]. It remains the foremost cause of kidney failure, lower limb amputations, and adult blindness. There are limited data on disparities in place of death of individuals with DM. This paper therefore seeks to enlighten on such disparities if they do exist.

Aims and objectives

The aim of this study is to evaluate the trends in places of death for mortality associated with DM in homes, hospice facilities, nursing homes, and medical facilities depending on age, gender, race, and census region in the United States from 1999 to 2020, and to describe the predictors of home or hospice death over nursing home or medical facility death for DM depending on age, gender, race, and census region in the United States from 1999 to 2020.

## Materials and methods

A retrospective study was conducted virtually in August 2023 using a freely accessible database available on the internet. Since no humans were involved, ethics committee approval was not needed.

The data for this study were extracted from the CDC WONDER (Centers for Disease Control and Prevention’s Wide-Ranging Online Data for Epidemiologic Research) database, a publicly accessible repository housing an extensive array of public health data. This repository includes information derived from U.S. death certificates since 1999. The database is searchable and encompasses details such as the location of death and specific patient demographics, which encompass factors such as age, gender, race, U.S. census region, and the causative factor behind the demise. These details pertain to patients whose healthcare providers have reported their deaths to the CDC. The data were acquired as of August 23, 2023.

For this investigation, the CDC WONDER database's Underlying Cause of Death segment was interrogated to identify individuals who passed away between 1999 and 2020. The collected patient attributes encompassed the year of death, place of death, age (grouped into 10-year ranges), race, U.S. census region (Northeast, Midwest, South, and West), and the root cause of death, categorized according to the International Classification of Diseases, 11th Revision (ICD-11) code range E10-E14. The places of death were classified into home/hospice versus alternative categories (such as inpatient medical facility, outpatient emergency medical facility, decease on arrival to a medical facility, unspecified medical facility, nursing home or long-term care facility, other places of death, or unknown place of death).

Statistical analysis was conducted utilizing the R programming software. univariate logistic regression was used as the analytical methodology. The future trends were predicted using the ARIMA model.

## Results

The aggregate data of 1,674,724 deaths over a 22-year period (1999-2020) was obtained for DM from the CDC WONDER database. These data can be found in Table 1 separated by death at home or hospice, a medical or nursing facility, or in other locations and by 10-year age groups, gender, census region, and race. Table 1 shows the absolute number of deaths at home or hospice, medical or nursing facility, and others based on age, gender, race, and census regions from 1999 to 2020. Maximum number of deaths at home or hospice were reported in age group 75-84 years (146,820), male gender (324,345), Census region 3: South (225,636), and white race (458,690) compared to their respective counterparts. In medical and nursing facilities, maximum number of deaths were reported in the age group of 75-84 years (305,482), females (538,783), Census region 3: South (401,645), and white race (805,863), compared to their respective counterparts. This trend can also be seen in the other 10-year age groups, with the most deaths occurring in a medical or nursing facility, followed by home or hospice and then other locations. A similar trend can be seen in the other patient identifiers. Both females and males show higher numbers of mortality in medical or nursing facilities at 538,783 and 502,819, respectively, as compared to 248,222 and 324,345, respectively, at home or hospice, or 27,565 and 32,990, respectively, in other locations. Separating patients by census region and race also demonstrates higher mortalities in patients in medical or nursing facilities, followed by home or hospice and then other locations.

**Table 1:** Total number of deaths at home or hospice, medical or nursing facility, and others based on age, gender, race, and census regions from 1999 to 2020.

	Home or hospice (n = 5,72,567)	Medical facility or nursing (n = 10,41,602)	Others (n = 60,555)
Ten-year age groups			
<1 year	0	42	0
1-4 years	15	96	0
5-14 years	100	553	29
15-24 years	1,563	2,120	538
25-34 years	6,093	8,140	1,179
35-44 years	18,126	24,218	2,586
45-54 years	52,049	71,074	5,639
55-64 years	107,989	153,796	9,462
65-74 years	142,595	238,405	11,773
75-84 years	146,820	305,482	14,894
85+ years	97,194	237,643	14,449
Gender			
Female	248,222	538,783	27,565
Male	324,345	502,819	32,990
Census Region			
Census Region 1: Northeast	91,196	188,611	6,056
Census Region 2: Midwest	121,691	255,248	12,347
Census Region 3: South	225,636	401,645	26,663
Census Region 4: West	134,044	196,098	15,489
Race			
American Indian or Alaska Native	72,70	11,858	884
Asian or Pacific Islander	16,125	28,695	16,43
Black or African American	90,482	195,183	10,161
White	458,690	805,863	47,867

Table 2 shows the statistical analysis of predictors of home or hospice death using univariate logistic regression to determine the odds ratio (OR) for each group and its statistical significance compared to a reference group for each patient identifier. In those over 85 years as reference, it was found that every age group outside of those between 1-4 had a statistically significant difference in mortality. A significantly high odds of death at home or hospice center was reported for the age groups of 55-64 years (OR: 1.76) and 45-54 years (OR: 1.754), male gender (OR: 1.381), Census region 4: West (OR: 1.352), and American Indian or Alaska Native (OR: 1.295). With females as reference, males had a statistically significant increase in mortality, with an OR of 1.381. When looking at census region and using patients in the Northeast as reference, every other census group had a statistically significant change in mortality, with those in the Midwest having a decreased mortality with an OR of 0.971 and those in the South and West having an increased mortality with an OR of 1.125 and 1.352, respectively. Finally, when looking at race and using Black or African American patients as reference, every other racial group had an increased mortality rate anywhere from 20.6% to 29.5%, with American Indian or Alaskan Natives being the most likely to die with an OR of 1.295.

**Table 2:** Statistical analysis of predictors of home or hospice death using univariate logistic regression to determine the odds ratio for each group and its statistical significance compared to a reference group for each patient identifier.

Variables	Univariate logistic regression		
	Odds ratio	95% confidence interval	P-value
Age			
1-4 years	0	(0, 598389.932)	0.441
5-14 years	0.405	(0.235, 0.698)	0.001
15-24 years	0.446	(0.36, 0.551)	<0.001
25-34 years	1.525	(1.432, 1.624)	<0.001
35-44 years	1.696	(1.641, 1.753)	<0.001
45-54 years	1.754	(1.719, 1.79)	<0.001
55-64 years	1.76	(1.736, 1.783)	<0.001
65-74 years	1.716	(1.697, 1.734)	<0.001
75-84 years	1.478	(1.464, 1.493)	<0.001
85+ years	1.0 (Reference)		
Gender			
Male	1.381	(1.372, 1.39)	<0.001
Female	1.0 (Reference)		
Census Region			
Census Region 1: Northeast	1.000 (Reference)		
Census Region 2: Midwest	0.971	(0.961, 0.981)	<0.001
Census Region 3: South	1.125	(1.114, 1.135)	<0.001
Census Region 4: West	1.352	(1.338, 1.366)	<0.001
Race			
White	1.219	(1.209, 1.23)	<0.001
American Indian or Alaska Native	1.295	(1.257, 1.334)	<0.001
Asian or Pacific Islander	1.206	(1.182, 1.231)	<0.001
Black or African American	1.000 (Reference)		

**Figure 1:**
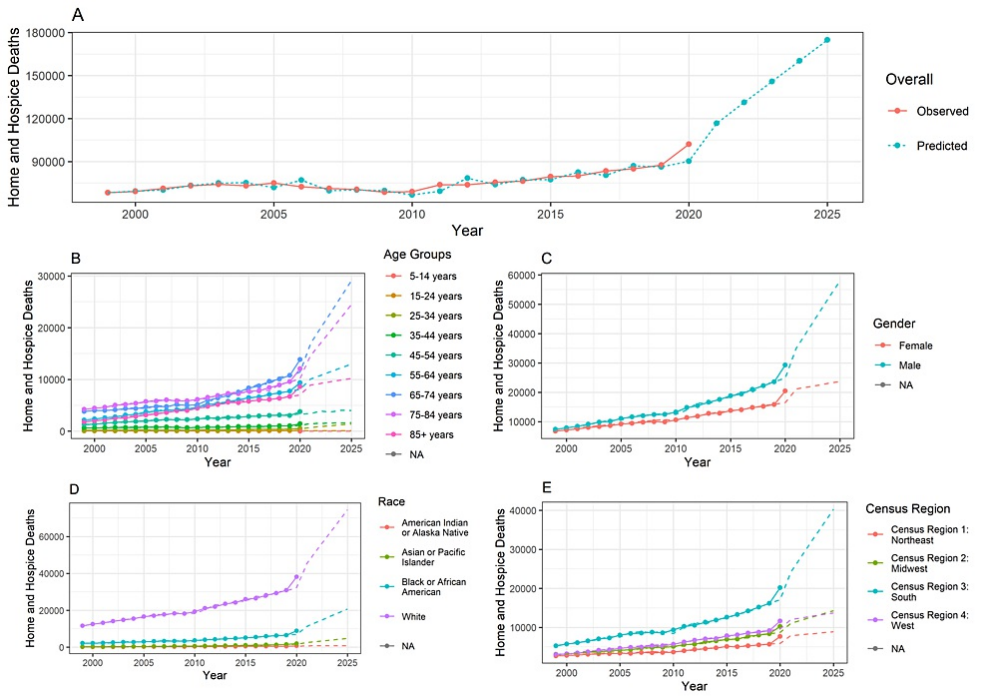
Home or hospice death trends. The forecasting is done from 1999 to 2025. The training data are available from 1999 to 2020. Therefore, the prediction is done for another five years. In the line chart, the lines represent the observed data, and the dotted line represents the forecasted data. The method used is the ARIMA model.

Figure 1A shows the trends in overall home and hospice deaths that have occurred from 1999 to 2024 due to DM, as well as predicted deaths from 1999 to 2025. Figures 1B-1E separate the data used in Figure 1a by 10-year age groups, gender, race, and census region. Each figure shows that the overall and predicted deaths at home or hospice have, for the most part, increased from 1999 to 2020 and are predicted to continue to increase up to 2025. Some groups, such as those in the age group of 15-24 years and American Indian or Alaskan Natives, showed very little increase in mortality over time, and their predicted mortality continues to follow this trend, but other groups showed that there has been an increasing number of mortalities at home or hospice over time and that mortality will continue to increase up to 2025. Of note, those aged 65-74 years, those aged 75-84 years, males, white, and those from the south showed marked increases in mortality from 1999 to 2020 and are predicted to continue to have large increases in mortality up to the year 2025.

## Discussion

This review and meta-analysis of the DM mortality trends used the CDC WONDER database over a 22-year period with four parameters (age, gender, census region, and race), which were extracted on August 23, 2023, to evaluate disparities in place of death in the United States.

According to the data, there were different mortality numbers for different places of death in the USA: there were 572,567 deaths in homes or hospices, 1,041,599 deaths at medical or nursing facilities, and 60,555 other deaths. The highest number of deaths occurred in medical and nursing settings, which were 62% of all deaths due to diabetes in different settings. For all causes of mortality trends, the home has surpassed the hospital as the most common place of death in the United States for the first time since the early 20th century [5]. When compared to England, hospitals continue to be a more prevalent place of all causes of death than home [6]; however, cumulative deaths due to diabetes in home or hospice settings in the United State are continuously increasing throughout recent years. The reason might be that death at home is preferred by most people, but for many people, this might not be possible or preferable [7].

Based on age, the highest number of deaths in home or hospice center were in the age group of 75-84 years (146,820), and followed by the age group of 65-74 years (142,595). This indicates that the mortality rate generally increases with age, except for the age group above 85 years, which may be due to a smaller base population number. This aligns with the common understanding that older age is associated with increased health risks. The mortality rate related to cardiovascular disease was significantly greater for those with long-term DM (17.3 per 1,000) than for those with freshly detected DM (11.5 per 1,000) [8]; as a result, as people age, their chances of developing complications from DM and the length of their condition also increase [9].

Males die more frequently at home, in hospice care, or from other causes, but females die more frequently in hospitals or nursing homes. Men who are in a relationship are more likely to be survived by their spouse due to their shorter life expectancy, which increases the likelihood that they will receive end-of-life care from family members at home. Other explanations for this can be biological variations, way of life decisions, and patterns of seeking healthcare. Increasing all-cause mortality was linked to DM in a recent meta-analysis; the relative risks were 1.59 for men and 2.00 for women [10], and women might be more likely to have diabetic complications that require hospital-seeking behavior.

Based on the census region, the highest number of deaths was in the Southern United States for home or hospice deaths, as well as for medical or nursing and for others. The West United States had the highest predictor of home or hospice death with an odd ratio of 1.352 (95% CI: 1.338-1.366). The Western U.S. is the most urbanized part of the country, which, according to the theory, in highly segregated neighborhoods, minority and low-income communities have better access to medical services via safety net providers and more targeted interventions [11-14]. This regional variation could be influenced by factors such as demographics, healthcare infrastructure, and economic disparities between different regions of the country.

Taking race variable into consideration, highest mortality was reported in white race for both home or hospice center (458,690) and hospital or nursing facility (805,863). American Indian or Alaska Native groups have the highest predictor of home or hospice death, with an odds ratio of 1.295 (95% CI: 1.257-1.334). Even though blacks consistently have worse health outcomes than whites do, certain research studies have demonstrated that in highly segregated areas, there are fewer differences among black and white race because black residents with low-income in high segregated areas have improved health conditions than low-income black residents in less segregated areas [11-15].

This study reports that the highest number of deaths at home and hospice center were found in the age group of 75-84 years, male gender, white race, and Census region 3: South, as compared to the respective counterparts. It is important to consider these factors when interpreting and analyzing such data to gain a comprehensive understanding of the trends and disparities observed.

Limitations

Some of the limitations of this study include the fact that the latest data from the years 2021 to 2023 were not included. This is because, as of yet, these data are unavailable on the CDC WONDER database. Furthermore, DM can be classified into several categories, some of which include type 1 DM, type 2 DM, gestational DM, and maturity-onset DM of the young. Future studies based on each category can be conducted when such data become available.

## Conclusions

There is an overall rising trend in home and hospice deaths in the United States for patients with DM, but with a steady dip between the years 2005 and 2010. Patient deaths from DM were categorized by age groups, gender, race, and census regions. The highest mortality trends were exhibited in whites, males, and those aged 75-84 years. Out of the census regions, the south has the highest mortality trend. Further studies could be carried out to determine the reasons for the rising trends in home or hospice deaths in the aforementioned groups and how to provide these groups with better access to healthcare facilities.
